# Chemical Cardioversion of Atrial Fibrillation with Calcium Gluconate

**Published:** 2012-10-30

**Authors:** A Serhat, Hayriye Gonullu, A Huriye

**Affiliations:** 1 Emergency Medicine Clinic, Izmir Bozyaka Training and Research Hospital, Izmir, Turkey; 2 Deparment of Emergency Medicine, Van Yuzuncuyil University, Van, Turkey

**Keywords:** Spinal epidural hematoma, Cord compression, Conservative therapy

## Abstract

**Background:**

Calcium infusion is used as a pre-treatment before calcium channel blockers to prevent hypotension. Occasional cardioversion with calcium gluconate infusion is seen in patients with paroxysmal supraventricular tachycardia. Several mechanisms have been suggested for mechanism. Herein we report a case presenting with atrial fibrillation but cardioverted with calcium gluconate infusion, which is unreported in the literature before.

## Introduction

Atrial fibrillation (AF) is the most common arrhythmia generated in the atrial tissue that overwhelms the normal electrical impulses generated by the sinoatrial node. It’s a frequently diagnosed arrhythmia in the emergency department (ED) which can lead to embolic cerebrovascular disease, congestive heart failure, or acute peripheral arterial occlussion. Synchronised cardioversion is the choice of treatment in unstable patients while rate control with β-blockers or calcium channel blockers (CCB) are preferred in stable patients before considering chemical cardioversion in selected patients.

Calcium infusion prior CCB in paroxysmal supraventricular tachycardia patients is used to prevent hypotension is supported by literature.(1,2) We report a case with AF who had cardioverted to normal sinus rhythm after infusion of calcium to prevent hypotension before infusion of CCB.

## Case Report

A 73 year old female patient admitted to ED with complaint of palpation that started few hours prior to admission. She denied chest pain, dyspnea, syncope and stated she didn’t have arrhythmia in the past. Her medical history included controlled hypertension with tiazid diuretic without any other identifiable cardiovascular risk factor. She denied taking any over-the-counter medication before admission.

Upon admission, her blood pressure was 93/68 mmHg with an irregular heart rate of 163/min with normal respiratory rate, axillary temperature and pulse oximetre. Her physical examination appeared normal except irregular tachycardic heart sounds and irregular pulses. Mental status was normal with full orientation and cooperation. Electrocardiogram revealed atrial fibrillation with rapid ventricular response without ST-T segment ischemic changes. Before administrating CCB diltiazem of 0.25 mg/kg (15 mg for 60 kg) for rate control, 10 ml of calcium gluconate infusion was slowly initiated to prevent hypotension which was followed by cardioversion to normal sinus rhythm. During follow-up, cardiac enzymes were within normal range and echocardiography conducted by cardiologist didn’t show thrombus. She was discharged with acetyl salicylic acid with recommendation of follow-up as a cardiology outpatient.

## Discussion

Atrial fibrillation is a frequently diagnosed arrhythmia in the ED.Treatment in unstable patients with chest pain, myocardial infarction, dyspnea, pulmonary edema, altered mental status and hypotension includes electrical cardioversion regardless of time of onset. In stable patients without unstable criterias mentioned above, if the time of onset is less than 48 hours electrical or chemical cardioversion can be tried depending on clinical status while rate control is preferred if the time of onset is more than 48 hours or unknown.

ß-blockers or CCB agents are used for rate control while digoxin is less preferred due to late onset of action. Most anticipated side effect of CCB is hypotension where it’s experienced less than β-blockers.(3) They are typically avoided in people who are hypotensive, or trending in that direction. Calcium has been used to prevent hypotension before verapamil-induced hypotension without compromising anti-arrhythmic effect in patients with paroxysmal supraventricular tachycardia (PSVT) is described in the literature.(4) It has been suggested primarily for prevention of hypotension, not for conversion of arrhytmias.

**Fig. 1:**
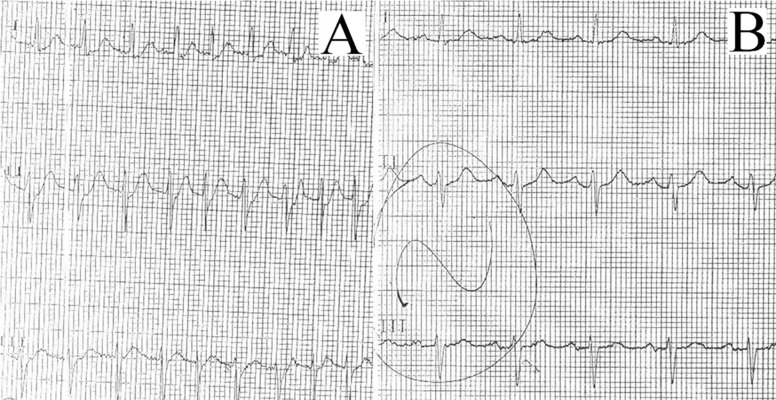
A) Presenting ECG as atrial fibrillation withrapid ventricular response. B) Rhythm converted to sinus after calcium gluconate infusion.

Cases presenting with PSVT cardioverted with calcium gluconate infusion are reported in the literature.(5) O'Brien et al. describes several cases of PSVT that were converted to normal sinus rhythm after receiving intravenous calcium infusion for prevention of hypotension after CCB. Authors proposed that calcium increases blood pressure by elevating parasympathetic tone, raising blood pressure and slowing atrioventricular conduction. Such an effect of intravenous calcium gluconate in an AF patient as in our case wasn’t described in the literature. Possible explanations of authors of previous cases probably fail to explain the distinct properties of our case but we propose that calcium overwhelms non-organized arrhythmogernic atrial nodes that generate atrial fibrillation by elevation parasympathetic tone.(Figure 1)

## Conclusion

Cardioversion effect of calcium in PSVT and AF patients, as in our case, needs to be evaluated in more studies to clarify its’ effect and whether it’s a causal or temporal effect. Clinicians more liberal use of calcium to prevent hypotension before CCB in PSVT or AF patients can increase the likelihood of cardioversion to normal sinus rhythm.
